# Development of New Effective Sorbents Based on Nanomagnetite

**DOI:** 10.1186/s11671-016-1371-3

**Published:** 2016-03-21

**Authors:** Dorota Kołodyńska, Marzena Gęca, Ievgen V. Pylypchuk, Zbigniew Hubicki

**Affiliations:** Department of Inorganic Chemistry, Faculty of Chemistry, Maria Curie Skłodowska University, M. Curie Skłodowska Sq. 2, 20-031 Lublin, Poland; Nanomaterials Department, Chuiko Institute of Surface Chemistry of National Academy of Sciences of Ukraine, 17 General Naumov Str., 03164 Kyiv, Ukraine

**Keywords:** Nanocomposite, Kraft lignin, Magnetite, Adsorption, Heavy metals

## Abstract

Magnetic hybrid nanocomposite material based on the kraft lignin was prepared by the co-precipitating method. Kraft lignin was modified by iron nanooxide in order to enhance its sorption properties towards heavy metal ions. The composite material was characterized by physicochemical methods such as BET N_2_, ATR-FTIR, TGA, DSC, pH_pzc_, XRD and SEM. Its adsorption behaviour was studied using the batch mode by varying different parameters like pH, initial concentration of metal ions and shaking time as well as the presence of interfering ions. Adsorption of Cu(II), Cd(II) and Pb(II) ions from the aqueous solutions was studied in comparison with the commercial kraft lignin. The adsorption capacity and kinetic sorption characteristics of the composite material were determined.

## Background

Due to heavy metal environmental persistence and intense utilization in several applications, development of cost-effective removal strategies is required for treating metal containing waters and wastewaters. A variety of well-known treatment methods exists for metal removal including precipitation, coagulation and co-precipitation, electrochemical treatment, ion exchange and membrane separation [1, 2]. However, high capital costs of the above mentioned processes led to explore more cost-effective options including the use of sorption media developed from such natural materials as biosorbents. Many low-cost adsorbents such as chitosan, clay, saw dust, lignin, pectin, seaweed, zeolite, bark materials and iron oxide-coated sand were previously investigated for metal ion removal. Lignin, the by-product of paper industry and emerging cellulose ethanol industry, is a potential metal sorbent.

Lignin is the second most abundant biopolymer, after cellulose on the earth. It exists naturally in all woody plants and is integrated into the plant cell wall. Type of tissues and cell wall layers as well as the stages and conditions of development determine the total content of lignin in any plant body, even within the same plant species. The three monolignol monomers that form lignin structure are: (a) *p*-coumaryl, (b) coniferyl alcohol and (c) sinapyl alcohol [3].
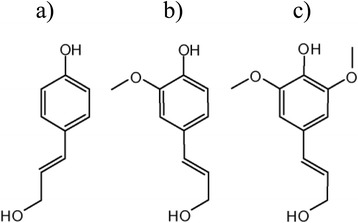


Most research related to lignin deals with the kraft lignin or black liquor of paper industry. Many other types of lignin were also designated as having dark black appearance. This is solely due to the self condensation of various functional groups of lignin macromolecules during separation from the lignocellulosic matrix. However, compared with polysaccharides, cellulose and hemicellulose, the effective use of lignin has not been progressed owing to the difficult separation of lignin from the other components of wood without their denaturation. The limited use of lignin compared to cellulose is attributable to its molecular structure; i.e. the lignin molecule lacks stereo-regularity and the repeating units of the polymer chain are heterogeneous. Also, lignin in wood forms interpenetrating polymer units with cellulose. Hence, using conventional methods, strong reagents are necessary to separate hydrophobic lignin from hydrophilic cellulose which results in the condensation of lignin on itself giving complicated molecular networks.

Recent industrial applications of lignin are mainly based on modified lignins. Their aromatic three-dimensional structure involving a large number of functional groups, such as methoxy groups, hydroxyl groups, carboxylic acid groups and sulfonate groups, suggests that these groups can play an important role in the formation of macromolecular materials. Therefore, the main aim of this study was to compare the relative ability of kraft lignin and modify by magnetite lignin to sorb Cu(II), Cd(II) and Pb(II) ions from aqueous solution in the batch technique. The batch method is mainly used for determination of physical and chemical properties of sorption: selectivity coefficients, stability of complexes in the phase of a sorbent, and its capacities. A secondary aim was to find the correlation between the sorption mechanisms and such important parameters as the surface properties (i.e., particle size, surface area, functional group composition/content) of selected lignin sorbents.

Modification of lignin by iron nanooxides was provided in order to enhance its sorption properties. Iron oxides have a relatively high surface area and effective adsorption groups. Various forms of iron oxides, e.g. magnetite Fe_3_O_4_, hematite α-Fe_2_O_3_ and maghemite γ-Fe_2_O_3_ are widely described in literature [2].

## Methods

### Materials and Apparatus

**Fig. 1:**
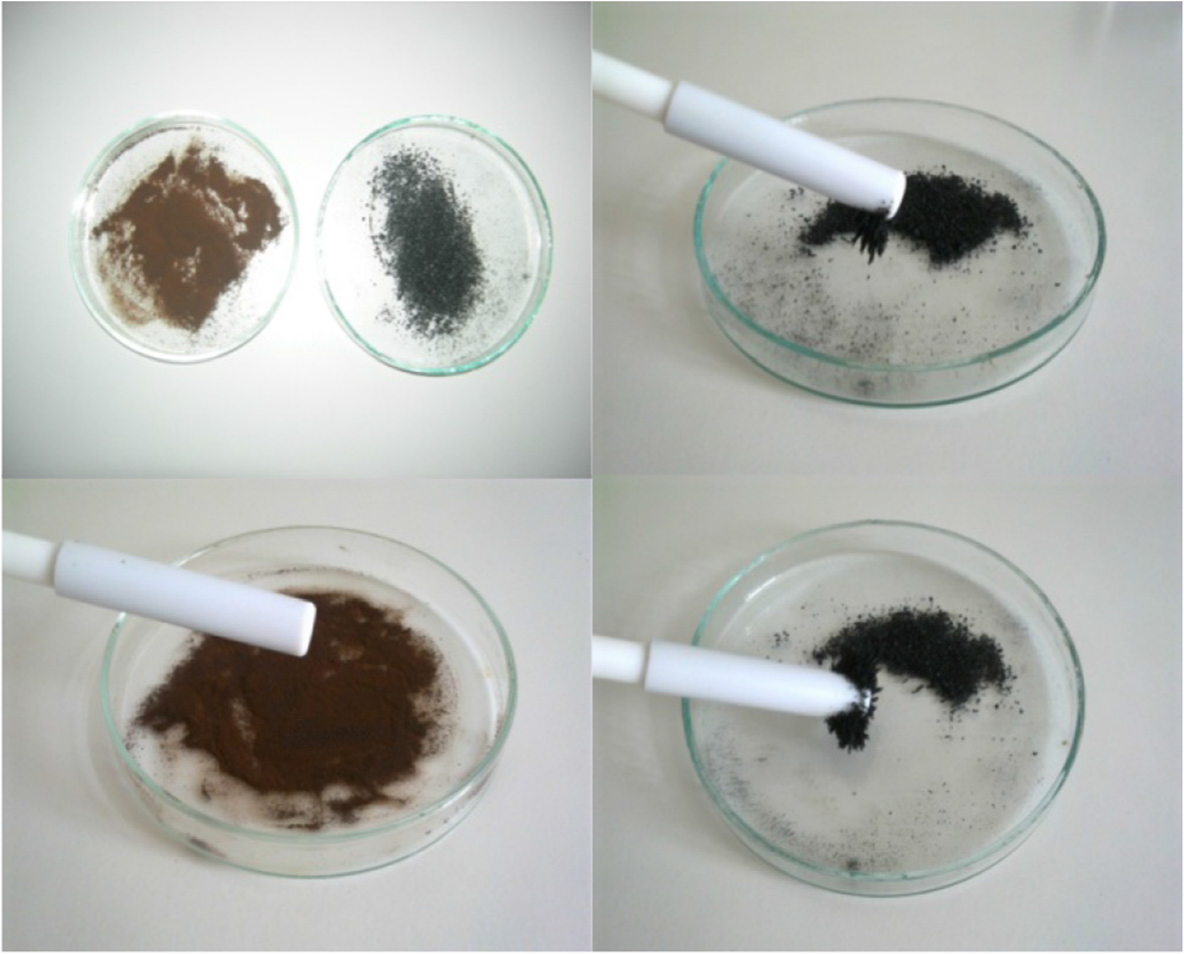
Kraft lignin before and after modification

Kraft lignin (Sigma-Aldrich) was modified by iron salts to obtain magnetic hybrid nanocomposite material according to the procedure described in [4]. Iron nanooxide was prepared by co-precipitating Fe(III) and Fe(II) ions by NaOH solution and treating under hydrothermal conditions. The synthetic products retained dark, black colour and strong magnetism, which are distinctive properties of magnetic iron oxides. The obtained sorbent is presented in Fig. 1. Chemical precipitation was achieved at 25 °C under vigorous stirring by adding 0.5 M NaOH solution till pH matched the value of 10.5. The precipitate was heated at 80 °C for 30 min, washed several times by deionized water and dried.

Aqueous solutions of Cu(II), Cd(II) and Pb(II) ions were prepared by dissolving corresponding analytical grade individual metal chlorides or nitrates. pH of the solutions was adjusted by dilute sodium hydroxide solution. The pH of the sample solution was measured by using a pH meter model M 85 Precision (Radiometer).

Batch experiments were carried out in triplicate in the pH range from 2–6, sorbent dose 0.01–0.5 g and the initial metal concentration 100 mg/dm^3^. A series of initial metal concentrations (*c*_0_) ranging from 25 to 100 mg/dm^3^ for kinetic analysis and from 25 to 500 mg/dm^3^ for isotherms, respectively, were prepared from metal stock solutions of Cu(II), Cd(II) and Pb(II). The initial suspension pH value was adjusted by adding 0.01 M HCl or 0.01 M NaOH.

The flasks were equilibrated at room temperature on a mechanical shaker (ELPIN+ 358 type, Elpin) with constant shaking at 180 rpm. Based on the preliminary tests, a 3-h equilibration time was chosen. After equilibration, the samples were filtered through 0.45-μm membrane filters (Millipore) and then analyzed for metal concentration by atomic absorption spectroscopy (AAS) using Spectr AA240 FS (Varian). All experiments were carried out in triplicate, and the deviation from the mean value was less than 5 % in all cases.

Thermal behaviour of kraft lignin was determined by thermogravimetric analysis (TGA) using Q50 TGA instrument. TGA measurements of 4–25 mg samples were carried out at 10 °C/min heating rate in the range of 25–1000 °C under nitrogen atmosphere with a flow rate of 50 cm^3^/min.

The differential scanning calorimetry (DSC) analysis was made using DSC TA Instruments Q20 equipped with a cooling system up to a temperature −90 °C; 2–10 mg samples were placed in the air-tight aluminum crucible and heated at the rate 10 °C/min in the range 25–450 °C under nitrogen atmosphere with a flow rate of 50 cm^3^/min. Measurements were made in the N_2_ atmosphere. DSC measures the exothermic and endothermic responses of the samples, while the FTIR analysis observes their changes in chemical and physical composition.

The Fourier transform infrared spectra of kraft lignin-based sorbents before and after the sorption process were registered using a Cary 630 ATR-FTIR instrument (Agilent Technologies) by the attenuated total reflectance technique.

The X-ray phase analysis (XRD) was made by the powder method using an X-ray diffractometer Philips X’pert APD (Panalytical) with a goniometer PW 3020 and Cu lamp a well as a graphite monochromator. The analyses were made at an angle 5–65 2*θ*.

The pH of the point of zero charge pH_zpc_ was measured using the pH drift method. The pH of the sorbent in the 0.01 M NaCl solution was adjusted between 2 and 12 by adding 0.01 M NaOH and 0.01 M HCl; 0.2 g of the adsorbent was added to 50 cm^3^ of the solution, and after 24 h, the final pH was measured.

All reagents were analytical grade and used without further purification. Demineralized water was used in preparation of all sample solutions (Hydrolab).

### Calculations

The adsorption amount (*q*_*t*_) and the sorption percentage (*S*%) were calculated according to Eqs. (1) and (2):

1 $$ {q}_t=\left({c}_{\mathsf{0}}-{c}_t\right)\times \frac{V}{m} $$

2 $$ S\%=\frac{c_0-{c}_t}{c_t}\times 100 $$

where *q*_*t*_ is the adsorption amount of heavy metal ion at time *t* (mg/g), *m* is the weight of sorbent (g), *V* is the volume of solution (dm^3^) and *c*_0_ and *c*_t_ are the initial and at time *t* concentrations of heavy metal ion in solution, respectively (mg/dm^3^).

Based on the obtained results, the distribution coefficients (*K*_*d*_, cm^3^/g) were also determined:

3 $$ {K}_d=\frac{c_{\mathsf{0}}-{c}_t}{c_t}\times \frac{V}{m} $$

where *c*_0_ and *c*_t_ are the initial and at time *t* concentrations of heavy metal ion in solution, respectively (mg/dm^3^), *V* is the volume of the solution (cm^3^) and *m* is the amount (g) of the sorbent.

When *t* is equal to the equilibrium contact time, *c*_*t*_ = *c*_e_, *q*_*t*_ = *q*_e_, and the amount of heavy metal ions adsorbed at equilibrium, *q*_e_, is calculated using Eq. (1).

Kinetic investigations usually apply the pseudo first order (PFO) and the pseudo second order (PSO) reaction models (Eqs. 4 and 5) [5, 6]:

4 $$ \log \left({q}_e-{q}_t\right)= \log \left({q}_e\right)-\frac{k_1t}{\mathsf{2}.\mathsf{303}} $$

5 $$ \frac{t}{q_t}=\frac{t}{q_e}+\frac{1}{k_2{q}_e^2} $$

where *q*_*e*_ is the amount of metal ion sorbed at equilibrium (also denoted as *q*_1_ and *q*_2_ for the PFO and PSO models, respectively) (mg/g), *q*_*t*_ is the amount of metal ion sorbed at time *t* (mg/g) and *k*_1_ and *k*_2_ are the equilibrium rate constants (1/min).

The comparison with another mass transfer model like the intraparticle diffusion one of Morris and Weber [7] is also presented:

6 $$ {q}_t={k}_i{t}^{\mathsf{1}/\mathsf{2}}+C $$

where *k*_*i*_ is the rate constant of diffusion (mg/g min^−1/2^) and *C* is the intercept*.*

The Elovich equation is one of the most useful models for describing chemisorption, which is given as [8]:

7 $$ {q}_t=\frac{1}{B}\left( \ln\ AB\right)+\frac{1}{B} \ln (t) $$

where *A* (mg/g min) is the initial sorption rate and *B* (g/mg) is related to the extent of surface coverage and activation energy for chemisorption (desorption constant).

Based on the linear plots of *q*_*t*_ versus ln *t*, the Elovich parameters can be calculated.

The adsorption data of metal ions on the kraft lignin-based sorbent were analyzed using the Langmuir and Freundlich isotherm models (Eqs. 8 and 9). The Langmuir model [9]:

8 $$ {q}_e=\frac{q_0{K}_L{c}_e}{1+{K}_L{c}_e} $$

where *c*_e_ is the equilibrium concentration of the metal ion (mg/dm^3^), *q*_e_ is the adsorption capacity at equilibrium (mg/g), and the constants *q*_0_ (mg/g) and *K*_*L*_ are the characteristics of the Langmuir equation (dm^3^/mg) which can be determined from its linearized form (plots of *c*_*e*_*/q*_*e*_ versus *c*_*e*_).

The Freundlich model [10]:

9 $$ {q}_e={K}_F{c}_e^{\mathsf{1}/n} $$

where *K*_*F*_ is the Freundlich adsorption capacity (mg/g) and *1/n* is the Freundlich constant related to the surface heterogeneity. The above equation can be linearized to calculate the parameters *K*_*F*_ and *n*.

## Results and Discussion

### Specific Surface Area

The measurement of specific surface area and average pore diameter was made using ASAP 2405 (Micrometrics Inc.). The specific surface area (*S*_BET_) was determined based on the Brunauer-Emmett-Teller (BET) multilayer adsorption. The total pore volume (*V*_*t*_) was determined from the adsorbed nitrogen volume at *p*/*p*_0_ = 0.99. The average pore diameter (*D*_*p*_) is estimated from the pore volume, assuming a cylindrical pore geometry and using the equation 4*V*_*t*_/*S*_BET_. The mesopore distribution curve was obtained from the adsorption branch of the N_2_ isotherm by the BJH method. The micropore distribution is calculated from the gas adsorption using the Horvath-Kawazoe equation, with relative pressure (*p*/*p*_0_) below 0.01.

**Fig. 2:**
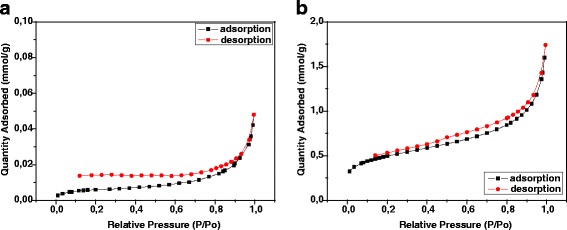
Nitrogen adsorption/desorption isotherms of the **a** lignin and **b** hybrid nanocomposite material

Figure 2 presents the nitrogen adsorption/desorption isotherms measured at 77 K for the kraft lignin and the magnetic hybrid nanocomposite material. Kraft lignin is expected to have a small surface area since the amounts of N_2_ adsorbed are very low. According to the results of surface area and average pore diameter analysis, kraft lignin has the BET surface area 0.43 m^2^/g and the average pore diameter 18.7 nm, whereas the magnetic hybrid nanocomposite material has the BET surface area 4.8 m^2^/g and the average pore diameter 20 nm. The specific surface areas of both materials are lower than that of the other well-known carbon-based sorbents (e.g., activated carbon) probably due to the lack of an extensive microporous structure in the lignin materials used in our study.

However, it is well known that lignin is characterized by the high carbon content 65 %, hydrogen content 4.2 % and low nitrogen content 0.01 %. Also, the high sulfur content 2 % originated from both the Kraft or sulfate process, based on the action of NaOH and Na_2_S for separating cellulose from the other wood constituents, can be also a great attribute for the lignin application in heavy metal ions removal even in the case of its low surface area [11]. In addition to the sulphur groups, the carbon containing carboxyl and hydroxyl groups of magnetic hybrid nanocomposite material can also be responsible for the sorption of M(II) ions. On the other hand, its sorptive properties can be improved by obtaining the hybrid material based on lignin and containing the iron oxide particles. There have been many methods of the synthesis of magnetite nanoparticles reported, such as reduction of hematite, co-precipitation of iron salts by ammonia solution, oxidation of iron gels, X-ray radiation, synthesis assisted by microwave radiation, and microemulsion [12]. The common way of magnetite synthesis is the alkaline hydrolysis of iron(II) and iron(III) salts. The size of formed particles depends on the relative oversaturation of solution, and the formation of nanoparticles is expected at very low and very high concentration.

**Fig. 3:**
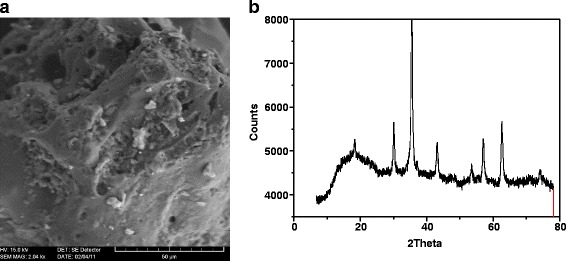
SEM image **a** and XRD patterns **b** of the hybrid nanocomposite material

Based on the SEM and XRD analysis, it was found that the obtained sorbent contains nanoparticles of magnetite (Fe_3_O_4_). In Fig. 3, the intensities of the characteristic peaks at 35.6° connected with this oxide are presented.

In solutions, magnetite can be in the cationic (FeOH_2_^+^) and anionic (FeO^−^) forms under acidic and alkaline conditions, respectively. For them, there have been the two protonation constants reported [13, 14]: FeOH_2_^+^ ⇄ H^+^ + FeOH, p*K*_*a,*1_ *=* 5.3 and FeOH ⇄ H^+^ + FeO^−^, p*K*_*a,*2_ = 8.8. The sorption of heavy metal ions on HFO could be explained by the formation of both monodentate and bidendate complexes between the heavy metal cations and the negatively charged species of iron oxide nanoparticles [14–17] as well as the reaction between the cation metal ions and the functional groups of kraft lignin. Therefore, the most important factor is determination of pH_zpc_ (point of zero charge) of the obtained composite.

### pH_zpc_ Analysis

**Fig. 4:**
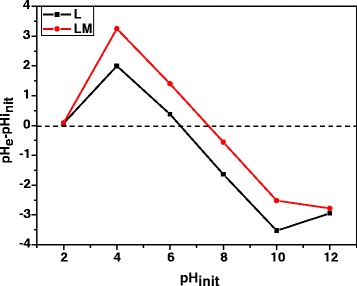
Determination of kraft lignin pH_pzc_ before and after modification by the drift method

The pH at which the charge of the solid surface is zero is referred to as the zero point of charge (pH_zpc_). At the solution pH values lower than pH_zpc_, the active sites of the sorbent are protonated and have positive charge. However, at the pH values higher than the pH_zpc_, the surface charge of the adsorbent is negative. In Fig. 4, the results of the pH drift method for determination of pH_zpc_ are presented.

It was found that pH_pzc_ of kraft lignin (L) is equal to 6.39. However, after modification by magnetite (LM), it is equal to 7.45. It can be assumed that the negative charge of active sites on the surface of the magnetic hybrid nanocomposite material allows metal ions or eventually metal hydroxides to be adsorbed on the surface.

### Effect of pH

The removal of Cu(II) using the magnetic hybrid nanocomposite material at different pH values in the range from 2 to 6 was studied using Cu(II) solutions at the concentration of 100 mg/dm^3^. It was found that the maximum removal (*S*%) was obtained at pH 6.0. With the increasing pH from 2 to 6, the residual concentration of Cu(II) decreases from 88 to 6.25 mg/dm^3^. Therefore, pH 6 was chosen as the more adequate value for the metal ion removal on the kraft lignin-based sorbents. For the metal ions, a similar variation of increase in *S*% with the increasing pH was observed. As the metal ions exist as cationic species at pH values less than 5.0, the removal takes place according to the mechanism of cation exchange. However, it should be mentioned that the most metal ions at pH higher than 6 can exist in the form of hydroxides. For example, for M(II) ions in the system, the dominant species is M(OH)_2_ and M(OH)^+^. At low pH particularly below pH_zpc_, the positively charged M^2+^ and M(OH)^+^ species present in the solution may exchange with H^+^ from –COOH or hydroxyl groups of carbon. It was found that the final pH of the solution is always less than the initial pH at pH range between 2.0 and pH of zero point charge.

### ATR-FTIR Analysis

**Fig. 5:**
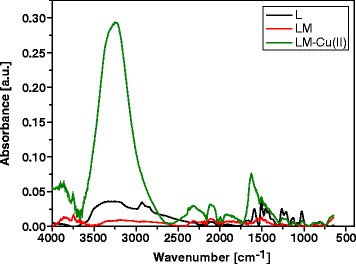
ATR-FTIR spectra of kraft lignin (*L*), magnetic hybrid nanocomposite material (*ML*) before and after the sorption process of Cu(II) ions (LM-Cu(II))

Chemical characterization of kraft lignin and lignin-based materials is not a simple task due to the three-dimensional structure, various chemical links and different functional groups. In addition, there are many different types of lignin depending on their original plants and processing methods [18]. The registered spectra of the studied sorbents before and after the sorption of Cu(II) ions are presented in Fig. 5.

It was found that after the sorption process, the broad and strong peak at 3344 cm^−1^ was due to the bound hydroxyl (–OH) groups. The peaks observed at 2933 cm^−1^ can be assigned to C–H stretching vibration, the CH_2_ stretching bands at 2919 and 2849 cm^−1^ are assigned to asymmetric and symmetric stretching of CH_2_ groups. The band at 1701 cm^−1^ of unmodified lignin can be attributed to the carbonyl group conjugated to the aromatic ring of lignin. The bands at 1590, 1455 and 1263 cm^−1^ are attributed to the C–O stretching of COOH groups, coupled OH in-plane deformation vibration and C(–O)–O stretching vibrations, respectively. The region between 1200 and 1000 cm^−1^ represented C–O stretching of alcohols and ethers. The bands at 1512 and 1117 cm^−1^ for unmodified lignin can be assigned to aromatic ring and C–O stretching of secondary alcohols, respectively [19]. In addition, common functional groups in lignin include methoxyl, phenolic hydroxyl, aliphatic hydroxyl and other carbonyl groups [20, 21]. After the sorption of Cu(II) ions, a strong band appears at 1628 cm^−1^.

### TGA and DSC Analysis

**Fig. 6:**
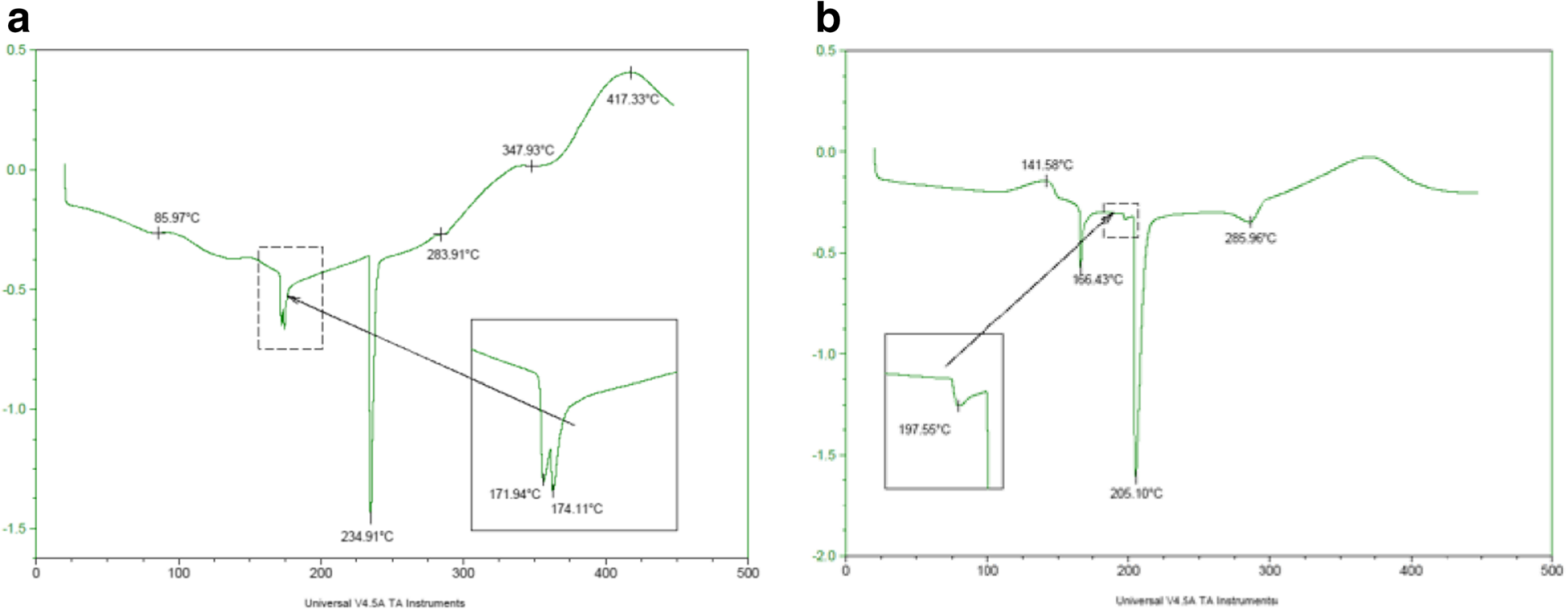
**a**, **b** TGA and DSC analysis of kraft lignin (*L*) and magnetic hybrid nanocomposite material (*ML*)

In Fig. 6a, the thermograms of kraft lignin analyzed by the DSC method are presented. There are six exothermic peaks in the DSC thermogram of the lignin at 85.977, 171.94, 174.11, 234.91, 283.9, 347.93 and 417.33 °C. Peak 2 is composed of two peaks at 171.94 and 174.11. As for the DSC thermogram of Fe_3_O_4_ (Fig. 6b), only four peaks are visible at 166.43, 197.55, 2015.1 and 285.96 °C.

### Effect of Shaking Time

**Fig. 7:**
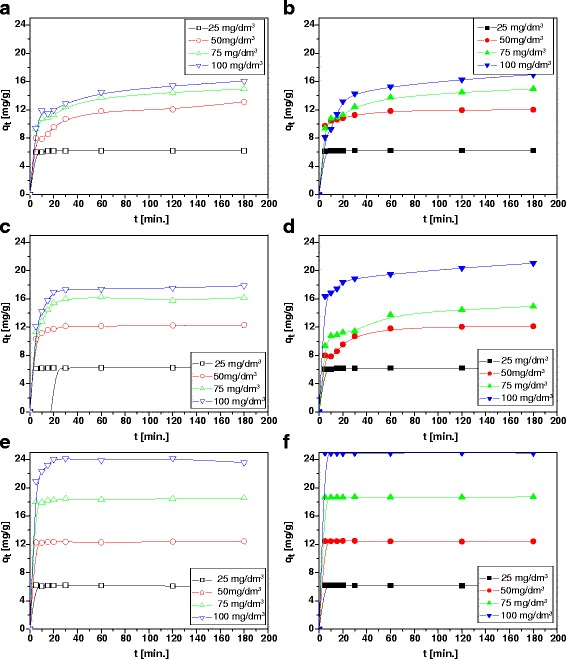
**a**–**d** Comparison of sorption capacities towards Cu(II) (**a**, **b**), Cd (**c**, **d**) and Pb(II) (**e**, **f**) on kraft lignin before (*open points*) and after (*solid points*) modification

The effects of shaking time on the sorption of Cu(II), Cd(II) and Pb(II) ions on kraft lignin as well as magnetic hybrid nanocomposite material are shown in Fig. 7. Based on them, it was found that the rate of their sorption was high at the beginning due to the accessibility of surface functional groups. More than 95 % of adsorption occurred within 30 min. However, the complete removal of selected metal ions under experimental conditions was achieved at shaking time 3 h. The percentage removal (*S*%) of metal ions(II) decreases with the increase of initial concentration. This can be explained by the fact that all adsorbents have a limited number of active sites which become saturated after certain concentration. It was proved that hybrid nanocomposite materials are characterized by slightly better sorptive properties than lignin. The obtained values of sorption percentage (%*S*) are as follows: 96, 99 and 98 % for Cu(II), Cd(II) and Pb(II), respectively, on kraft lignin and 99, 97 and 98 %, respectively, on the magnetic hybrid nanocomposite material. Moreover, *K*_*d*_ values (log *K*_*d*_) for these ions are equal to 4.83, 3.44 and 2.37 cm^3^/g and 4.95, 4.65 and 2.65 cm^3^/g, respectively.

### Effect of Initial Concentration of Metal Ions

In Fig. 2a–f, plots of the sorption of Cu(II), Cd(II) and Pb(II) ions on the kraft lignin and the magnetic hybrid nanocomposite material at initial concentration 25, 50, 75 and 100 mg/dm^3^ at different time intervals were presented. The pH of the systems was maintained at 6.0.

Based on them and the plots of log(*qe − qt*) versus *t* and *t/q*_*t*_ versus *t*, the sorption parameters were determined. The exemplary results for the determined parameters for Cu(II) ions removal on kraft lignin and Pb(II) ions on the magnetic hybrid nanocomposite material are presented in Tables 1 and 2. Contrary to the PFO, the fitting of the kinetic data in the PSO equation showed excellent linearity with the high determination coefficient (*R*^2^) equal to 1.0000 in the concentration range 25–200 mg/dm^3^ for Cu(II), Cd(II) and Pb(II).

**Table 1:** Kinetic parameters of Cu(II) ions removal on kraft lignin for the initial concentrations 25, 50, 75 and 100 mg/dm^3^

Parameters	Cu(II) ions concentration			
	25 mg/dm^3^	50 mg/dm^3^	75 mg/dm^3^	100 mg/dm^3^
PFO				
*q*_e,exp_	6.21	14.49	18.70	24.93
*q*_1,cal_	0.11	0.09	0.06	0.05
*k*_1_	0.023	0.019	0.014	0.013
*t*_1/2_	0.211	0.203	0.248	0.258
*R* ^2^	0.6923	0.9829	0.9429	0.8994
PSO				
*q*_2,cal_	6.23	12.49	18.71	24.96
*k*_2_	1.198	0.852	1.200	1.309
*h*	46.510	132.999	420.222	815.470
*t*_1/2_	0.192	0.068	0.064	0.052
*R* ^2^	1.0000	1.0000	1.0000	1.0000
IPD				
*k*_i1_	0.02	0.02	0.06	0.006
*k*_i3_	0.008	0.01	0.007	0.004
*k*_i3_	0.004	0.004	0.004	0.004
*R*_i,1_^2^	0.9791	0.6157	0.9442	0.9213
*R*_i,3_^2^	0.9508	0.9782	0.9535	0.8997
*R*_i,3_^2^	1.0000	1.0000	1.0000	1.0000
Elovich				
*A*	7.35	4.63	3.35	2.31
*B*	53.82	37.94	55.20	71.17
*R* ^2^	0.9992	0. 9992	0.9768	0.8692

**Table 2:** Kinetic parameters of Pb(II) ions removal on magnetic hybrid nanocomposite material for the initial concentrations 25, 50, 75 and 100 mg/dm^3^

Parameters	Pb(II) ions concentration			
	25 mg/dm^3^	50 mg/dm^3^	75 mg/dm^3^	100 mg/dm^3^
PFO				
*q*_e,exp_	6.23	14.13	14.96	16.06
*q*_1,cal_	0.07	4.91	5.33	5.87
*k*_1_	0.017	0.021	0.019	0.015
*t*_1/2_	0.259	0.003	0.004	0.003
*R* ^2^	0.9487	0.8207	0.9786	0.9814
PSO				
*q*_2,cal_	6.21	13.36	15.31	16.43
*k*_2_	0.797	0.410	0.121	0.010
*h*	3.774	1.840	2.683	2.622
*t*_1/2_	0.128	0.111	0.031	0.001
*R* ^2^	1.0000	0.9976	0.9994	0.9990
IPD				
*k*_i1_	0.09	0.34	0.96	1.33
*k*_i3_	0.06	0.36	0.47	0.52
*k*_i3_	0.004	0.438	0.199	0.272
*R*_i,1_^2^	0.9791	0.9922	0.9751	0.9999
*R*_i,3_^2^	0.9508	0.7985	0.9007	0.9406
*R*_i,3_^2^	1.0000	1.0000	1.0000	1.0000
Elovich				
*A*	43.12	48.84	64.65	92.01
*B*	0.72	0.61	0.60	0.53
*R* ^2^	0.9867	0.9192	0.9798	0.9838

Moreover, the values of *k*_2_ are greater than *k*_1_ values and decrease with the increasing initial concentration. According to the Weber and Morris model, it is possible to establish whether the sorption is the intraparticle diffusion or not. It was found that the plots of *q*_*t*_ versus *t*^1/2^ do not pass through the origin. The multilinearity in the shape of the intraparticle diffusion plots has also been observed. The results indicate that the intraparticle diffusion rate decreased with the increase of the initial concentration from 25 to 100 mg/dm^3^. As for the Elovich model, it was found that it is more suitable for lower concentrations.

As for sorption parameters, the values of *q*_*o*_ and *K*_*L*_ were calculated from the intercept and slope of the linear plots of *c*_*e*_*/q*_*e*_ versus *c*_*e*_. The monolayer capacities for the kraft lignin are as follows: 56.11 mg/g for Cu(II), 63.98 mg/g for Cd(II) and 101.33 mg/g for the Pb(III). Therefore, the determined affinity series of metal ions for lignin is as follows: Pb(II) > Cd(II) > Cu(II). The same tendency was observed in the case of magnetic hybrid nanocomposite.

The process efficiency is also affected by the presence of NO_3_^−^ and SO_4_^2−^ ions at the initial concentration 100 mg/dm^3^; practically 87 % of the Cu(II), Cd(II) and Pb(II) ions is removed.

Magnetite lignin-based composites are quite a new type of sorption materials despite the fact that the literature describes application of this type of materials as catalysts. Lignin has also been recently used for the production of activated carbons by thermal treatment in the presence of alkali metal hydroxides such as NaOH and KOH [22–24]. The sorbent preparation procedure involves phosphorylation of lignin with phosphoric acid in the presence of urea as described by Bykov and Ershov [25]. The sorbent has a maximum possible phosphorus content and exhibits high sorption activity for Cu(II) and U(VI). The total exchange capacity was 0.86 mmol/g for Cu(II) ions and 0.23 mmol/g for UO_2_^2+^. In the paper by Li et al. [26], biochars from lignin, cellulose and wood were prepared at 400 and 600 °C. Authors suggest that due to excellent sorption capacities, lignin biochars exhibited a sorption capacity >180,000 mg/kg comparable to both cellulose and wood biochars. They are good candidates for uptake of aromatic pollutants. A magnetic lignin composite was prepared and modified with diethylenetriamine and described in [27]. Its sorptive properties were tested towards Cr(VI). Based on the Langmuir isotherm model, the calculated maximum Cr(VI) capacity *q*_m_ was equal to 123 mg/g. Regeneration of the magnetic lignin composite was achieved by 0.4 M NaCl and 0.2 M NaOH solution, and more than 87 % efficiency of desorption was obtained after 5 cycles.

### Stability Studies

**Fig. 8:**
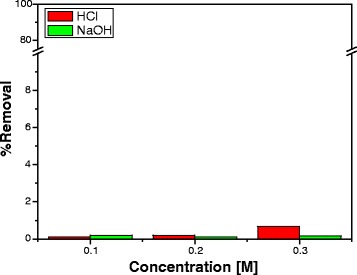
Comparison of %*R* of Fe(III) ions from the magnetic hybrid composite using HCl and NaOH are different concentrations

Lignin is a complex polymer of phenylpropane units, which are cross-linked to each other with a variety of different chemical bonds. It is well known that due to its complex composition and structure, the degradation of lignin is strongly influenced by its nature, reaction temperature, heating rate, degradation atmosphere and used solvents. Biodegradability can be enhanced by acids, bases, ammonia and urea as well as grinding and milling, fungal degradation or combined base and heat treatment. Moreover, some organisms, particularly fungi, have developed the necessary enzymes to break lignin molecules. As follows from the preliminary results, in acidic and base solutions, lignin composite is stable as can be seen in Fig. 8.

Moreover, in the paper by Mostashari et al. [28], the use of Fe_3_O_4_ magnetic nanoparticles as a recoverable adsorbent for lignin removal from aqueous solutions has been investigated. They tested several sorbents for magnetite particles regeneration. Desorption of lignin from Fe_3_O_4_ was studied using deionized water and different kinds of organic solvents, including acetonitrile, ethanol and methanol. According to the results, the desorption ability of acetonitrile was found to be the highest of all the solvents. The results showed that a desorption efficiency above 80 % can be achieved in a short time of 10 min and in a one-step elution using 5 mL of acetonitrile. The recovery for deionized water, EtOH and MeOH was lower than 40, 50 and 65 %, respectively. However, it should be mentioned that acetonitrile is quite expensive and not used in the wastewater treatment.

## Conclusions

Based on the results obtained, both lignin and modified lignin can find use in the removal of heavy metal ions such as Cu(II), Cd(II) and Pb(II). The pH dependence of used sorbents may suggest that the metal ions are adsorbed according to ion exchange and chelating mechanisms. The main parameters affecting sorption are initial concentration of the solution, pH and phase contact time. Temperature has only a slight influence. With respect to effectiveness of removal of Cu(II), Cd(II) and Pb(II) on the obtained hybrid sorbents, it can be arranged as follows: modified lignin by nanoiron oxides > lignin without modification.
